# Severe Gastrointestinal Haemorrhage: Summary of a National Quality of Care Study with Focus on Radiological Services

**DOI:** 10.1007/s00270-016-1490-3

**Published:** 2016-11-10

**Authors:** Simon J. McPherson, Martin T. Sinclair, Neil C. E. Smith

**Affiliations:** 1 Department of Radiology, Leeds Teaching Hospitals Trust, B Floor, Jubilee Wing, Great George Street, Leeds, LS1 3EX UK; 2 NCEPOD (National Confidential Enquiry into Patient Outcome and Death), Abbey House, 74-76 St John Street, London, EC1M 4DZ UK; 3 Dept of Surgery, Ipswich Hospital NHS Trust, Heath Road, Ipswich, IP4 5PD Suffolk UK

**Keywords:** Gastrointestinal, Haemorrhage/haemorrhage, Quality of care, Blood transfusion, Endoscopy, CT, Interventional radiology services (clinical practice), Embolisation, Surgery

## Abstract

**Purpose of Study:**

To identify the remediable factors in the quality of care provided to patients with severe gastrointestinal (GI) bleeding.

**Method:**

All hospital admissions in the first four months of 2013 with ICD10 coding for GI bleeding who received a transfusion of 4 units or more of blood. Up to five cases/hospital randomly selected for structured case note peer review. National availability of GI bleeding services data derived from organisational questionnaire completed by all hospitals.

**Results:**

4563/29,796 (15.3%) of GI bleeds received 4 or more units of blood with a mortality rate of 20.2% compared to 7.3% without blood transfusion. 30.8% of GI bleeds received a blood transfusion. 32% (60/185) of hospitals admitting acute GI bleeds lacked 24/7 endoscopy. 26% (48/185) had on-site embolisation 24/7 with a further 34% (64/185) accessing embolisation by transfer within a validated formal network. Blood product use was inappropriate in 20% (84/426). Improved management, principally earlier senior gastroenterologist review and/or endoscopy, would have reduced blood product use in 25% (113/457). 14.5% (90/618) had a CT scan which identified the site of bleeding in 32% (29/90). 7.8% (36/459) underwent an Interventional Radiology (IR) procedure but a further 6.3% (21/33) should have had IR. 6% (36/586) underwent surgery with 21/36 for uncontrolled bleeding. In 20/35 IR was not considered despite the majority being suitable for IR. Overall 44% (210/476) received an acceptable standard of care according to peer review.

**Conclusions:**

26 recommendations were made to improve the quality of care in GI bleeding, with six principle recommendations.

## Introduction

The diagnosis and treatment of gastrointestinal (GI) bleeding is challenging due the long length of the GI tract, the wide range of potential pathologies, the intermittent nature of bleeding and its occurrence in patients with multiple co-morbidities which may restrict the diagnostic and therapeutic options.

Traditionally, GI bleeding is split into upper GI (proximal to the ligament of Treitz, the limit of reach of a standard upper endoscope) and lower GI bleeding with management by medical and surgical teams, respectively. Lower GI bleeding is three times less common than upper GI bleeding [1]. Interventional radiology (IR) is established as the second-line intervention for upper GI bleeding when oesophago-gastro-duodenoscopy (OGD) fails to control bleeding and the first-line intervention in lower GI bleeding when medical management is ineffective [2, 3].

GI bleeding is the second commonest medical reason for transfusion in the UK after haematological malignancy, accounting for 14% of all blood transfusions [4].

Around 15% of upper GI bleeds occur in patients who are already in hospital and these are associated with higher mortality rates [5]. There are no comparable data available for lower GI bleeding. The significance of this is that the burden of caring for patients with a GI bleed, at least in the initial phase of their illness, may fall to any medical team, ward or hospital.

This paper reports selected findings and recommendations from a quality of care study “Time to get control” published by the National Confidential Enquiry into Patient Outcome and Death (NCEPOD^1^) 2015 with focus on the findings related to radiology services [6].

## Purpose

The purpose of the study was “To identify the remediable factors in the quality of care provided to patients with severe GI bleeding”.

## Method

### Study Population and Design

All patients who were admitted to hospital in the 4 months between 1 January 2013 and 30 April 2013 and who had a diagnosis of GI bleeding by ICD10 coding at any point during their in-patient stay were identified to NCEPOD. Local blood transfusion data were used to identify a sub-population of patients who received 4 or more units of red blood cells for their GI bleed. Cases were then selected for detailed review with a maximum of five cases per hospital. This established NCEPOD methodology allows quality of care in lower volume units to be assessed. This strength is offset by the potential to skew quantitative outcome data such as mortality within the study population. This limitation does not apply to the total population data.

Data were derived from three sources

- An *organisational questionnaire* completed by all hospitals included information on admission location, endoscopy services, interventional radiology services, surgical services, guidelines and standard operating procedures relevant to the management of GI bleed patients.

For each patient selected for the study

- A *clinician questionnaire*. Completion was led by the consultant responsible for the patient and included the patient’s presenting features/co-morbid conditions, initial management, investigations/procedures carried out, treatment, complications and escalation of care.
- Structured *review case note review*. A multidisciplinary group of expert peer reviewers from gastroenterology, acute medicine, interventional radiology and surgery reviewed a full copy of the admission case notes with round table discussion. The case reviewers answered a number of specific questions by direct entry into an electronic database with free text commentary and structured grading of care.

The denominator varies in the presented data depending on its source and if the question could be answered.

### Study Findings

#### Total Population

31,412 patients were identified by ICD10 coding as suffering a GI bleed of any severity in the 4-month study period, equating to an annual incidence of around 100,000 GI bleeds per year in the UK.

Blood transfusion and outcome data were recorded for 29,796/31,412 (Table 1). The overall mortality rate in this unselected GI bleed population was 10.4%. Patients requiring no blood had the lowest mortality rate of 7.3%. Approximately, a third of patients (30.8%) received a transfusion of one or more units of blood. Mortality increased with number of units of blood received. One in five patients who received 4 units or more of blood died. It might be presumed that this is simply to be due to the severity of the GI bleed, but blood transfusion has adverse effects and unnecessary transfusions cause harm. The place of restrictive transfusion protocols in GI bleeding continues to be evaluated [7].

**Table 1:** ICD10 coding, transfusion and mortality data from England, Wales and Northern Ireland for the first 4 months of 2013

	Total number of patients	%	Morality (%)
All patient	29,796		10.4
No blood	20,631	69.2	7.3
1–3 units	4602	15.5	14.6
≥4 units	4563	15.3	20.2

#### Study Population

618 (80%) of the 769 requested clinician questionnaires were returned. 485 case notes were sufficiently complete to allow peer review.

#### Organisation of Services

The majority (91.6% 186/203) of hospitals admitted patients with acute GI bleeding. 88% of hospitals had formal Hospital Guidelines for the management of upper GI bleeding. Planning for the treatment of lower GI bleeding care was much poorer with only 25% having formal guidelines.

National Quality Standards recommend that OGD should be available within 2 h of resuscitation for patients with an upper GI bleed which causes haemodynamic instability and should be performed within 24 h of presentation for all acute GI bleeds [8]. Despite this guidance, 32.4% (60/186) of hospital admitting patients with GI bleeding could provide 24/7 access to OGD. 23/60 had attempted to ameliorate the deficiency in their services by establishing a formal network. The recognition of a formal network by the referring and receiving hospital was verified by NCEPOD with no deficiencies found. Some patient’s will not be fit enough for transfer and formal networks will not address the needs of all patients, but the situation is even more parlous in the 20% (37/186) of hospitals who admit patients with GI bleeds and have no on-site or formally networked 24/7 cover.

One third of all hospitals 33% (67/202) had an IR service on-call. 27% (56/202) had an interventional radiologist on-call rota which could provide GI bleed embolisation on-site 24/7. This was validated by NCEPOD by checking the provided competency list for each individual on the rota. However, when the availability of a vascular radiographer and radiology nurse was included, a further eight hospitals had an incomplete service. Overall, 26% (48/185) of hospitals who admitted GI bleeds could offer embolisation 24/7.

Further, 32% (64/199) of all hospitals and 34% (64/185) of hospitals admitting acute GI bleeds were part of a formal network for embolisation of GI bleeding. Those sites with no on-site IR were more likely to be in a formal network than those that had a partial (i.e.,not 24/7) service. As with the OGD networks, these networks were validated. The 64 hospitals coupled with the 48 hospitals that had an on-site 24/7 service equates to only 56% (120/199) of hospitals being able to provide a 24/7 service for embolisation either on-site or by inter-hospital transfer.

Transjugular intrahepatic portosystemic shunt (TIPSS) was available 24/7 in 6% (13/205) of hospitals. Half of the remainder (51%, 94/185) of hospitals were part of a formal network to address the deficiency in on-site services. Whilst temporary tamponade with a Sengstaken or similar tube can mitigate the need for overnight TIPSS, a daily service needs to be available for patients with variceal bleeding.

97% (172/177) of hospital who admitted patients with GI bleeding had on-call surgical services on-site which could manage a GI bleed 24/7.

#### Admission

Almost all hospitals which admitted patients thought to have upper GI bleeds under the care of gastroenterologists, acute/general physicians or hepatologists and those thought to have lower GI bleeds under general or colorectal surgeons.

37.8% (180/476) of the patients in the study had a GI bleed complicating an admission for another condition. 97% of these admissions were non-elective. Severe GI bleeding appears to complicate patients admitted as an emergency with another condition rather than complicate elective admissions (Table 2).

**Table 2:** Presentation of GI bleed—New admissions versus established in patients

Presentation	Number of patients	%
Admitted with a GI bleed	296	62.2
Inpatient GI bleed	180	37.8
Subtotal	476	
Unknown	9	
Total	485	

#### Presentation

80% (164/205) of hospitals admitted upper GI bleeds to multiple locations (excluding level 2/3 care). Nursing experience in managing GI bleeds will be reduced by such a policy. Lower GI bleeds were more likely to be admitted to a single location (general surgical ward or surgical assessment unit).

**Fig. 1:**
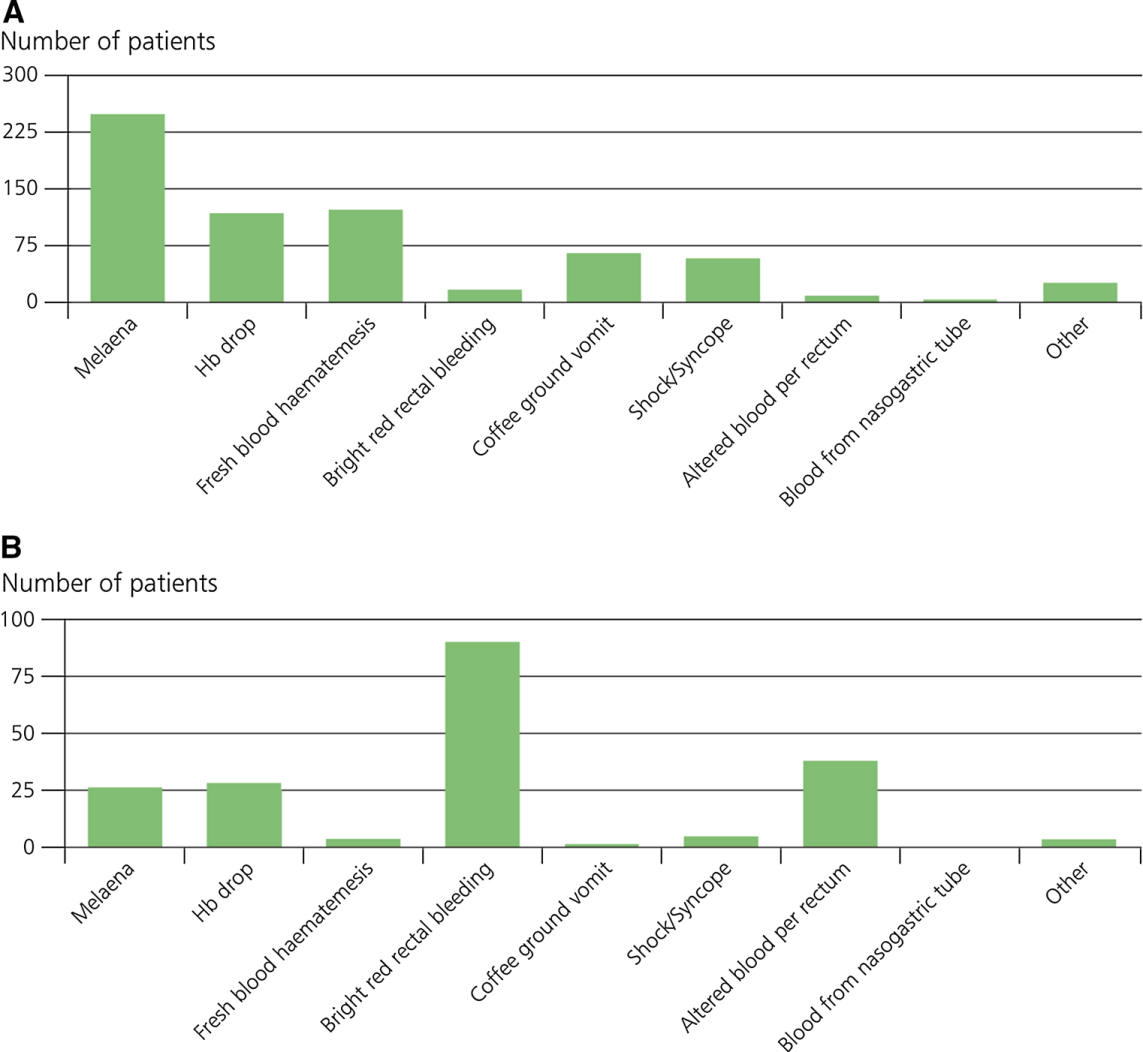
**A** Features of upper GI bleeds. **B** Features of lower GI bleeds

Figure 1A and B show the presenting features for those with patients where an upper or lower GI site of bleeding was confirmed.

This shows considerable overlap between the presenting features versus the eventually identified site of bleeding. This means that teams who think they are managing an upper GI bleed are actually managing lower GI bleeding and vice-a-versa. Bright red rectal bleeding and fresh haematemesis were the only presentations with low numbers of overlap (7.5 and 8% respectively), but no presentation was 100% predictive for the type of GI bleed.

In 21% (35/170) of patients who developed a GI bleed, whilst an in-patient for another condition, the reviewers identified clinically significant delays in recognition of the GI bleed despite clear signs suggestive of a GI bleed recorded in the notes. Deficiencies were commonest in established in-patients with another condition, particularly when clinical assessments were made by trainees from specialities who did not routinely manage GI bleeds.

#### Severity of Bleed at Presentation

A number of risk assessment scores exist for upper GI bleeding at the time of presentation (e.g., Blatchford, clinical Rockall and Glasgow). Despite NICE guidance recommending their use, only 34.1% (125/367) had an initial risk assessment score recorded. There are no widely adopted scoring systems for lower GI bleeding.

From the haemodynamic measurements provided at the time of presentation with a GI bleed, the NCEPOD researchers calculated the shock index (systolic blood pressure/heart rate) for each patient (Table 3). 12% (73/610) were identified in the clinical questionnaire as having haemodynamic compromise (shock or syncope). A shock index of >1 indicates haemodynamic compromise. 26% (152/587) had a shock index of >1 suggesting deficiencies in the clinical recognition of haemodynamic compromise. A shock index of one or greater has been shown to be predictive of active bleeding at imaging [9].

**Table 3:** Ranges of shock index (systolic blood pressure/heart rate) at presentation

Shock index	Number of patients	%
≤0.7	210	35.8
>0.7 ≤ 1	225	38.3
>1 ≤ 1.3	101	17.2
>1.3	51	8.7
Subtotal	587	
Insufficient data	31	
Total	618	

The outcomes relative to shock index ranges are shown in Table 4. This shows a rising mortality with increasing shock index at presentation.

**Table 4:** Mortality relative to shock index at presentation

Shock index	Alice	Deceased	Morality (%)	Total
≤0.7	172	38	18.1	210
>0.7 ≤ 1	170	55	24.4	225
>1 ≤ 1.3	73	28	27.7	101
>1.3	36	15	29.4	51
Insufficient data	25	6	19.4	31
Total	476	142		618

#### Diagnosis

The prevalence of different categories of GI bleeding is shown in Table 5. Despite requiring a blood transfusion of at least 4 units of blood, 11.7% (72/618) never had a cause of GI bleeding being identified.

**Table 5:** Type of GI bleeding from clinical questionnaire

Type of GI bleed	Number of patients	%
Non-variceal upper GI bleed	358	57.9
Lower GI bleed	138	22.3
Variceal upper GI bleed	50	8.1
Not diagnosed	72	11.7
Total	618	

#### Use of Blood Products

100% of hospitals had a massive transfusion protocol. The case reviewers considered that the use of blood products was appropriate in 80.3% (342/426). In 24.7% (113/457), improved clinical management could have reduced the use of blood products. The commonest cause was a delay to OGD which accounted for 35% (39/113).

#### Oesophago-Gastro-Duodenoscopy (OGD)

The majority of patients had (79%, 490/618) an OGD. Table 6 shows the findings at OGD. 26 patients had an OGD inappropriately omitted. The reasons were equally split between an on-site service or delayed access to an on-site service and delayed ward referral or the patient being considered “too unwell” for OGD. Over half (58%, 80/137) of the patients with lower GI bleeding had an OGD. In many of these, the reviewers commented in free text that alternative investigations should have been performed first and that the OGD could have been avoided. In 31% (114/369) cases, the time to OGD was too long for the patient’s condition. This was less of an issue (8.5%) where the first consultant review was by a clinician with responsibility for GI bleeding (gastroenterologists, hepatologists, colorectal or general surgery).

**Table 6:** The findings at OGD

Findings at OGD	Number of patients	%
Non-variceal bleeding	213	46.1
Variceal bleeding	38	8.2
Upper GI bleeding but cause obscured by blood	25	5.4
No upper GI bleed found	186	40.3
Subtotal	462	
Not answered	28	
Total	490	

174 patients had endoscopic treatment for non-variceal upper GI bleeding with the endoscopic management considered appropriate in 89% (154/174). The commonest reason for inadequate treatment was the use of adrenaline monotherapy which is known to be associated with higher rates of re-bleeding (2). 92% (35/38) of patients with variceal bleeding had endoscopic therapy with 31 having the NICE recommended treatment for oesophageal varices of band ligation (2). No patient had isolated gastric variceal bleeding in this study. Variceal bleeding was controlled at the first endoscopy in 65 % (25/38). Four patients required a Sengstaken or similar tube.

Despite deficiencies in monitoring, including omission of pulse oximetry or blood pressure recording in 23.9% (66/276), and this being a particularly unwell cohort, the complications of OGD were low at 2.2%

#### CT Scanning

A CT scan to diagnose the site of GI bleeding was performed in 14.5% (90/618). A further 20 should have undergone a CT. The CT identified the site of bleeding in 32% (29/90). This real world figure is considerably lower than the reported meta-analysis pooled literature sensitivity of 89% [10]. Whilst comprehensive data on the timing of CT were not readily available to the reviewers, their free text comments suggested that the much lower diagnostic rate was related to referrals for CT after the patient had been resuscitated and had clinically stopped bleeding or delays in performing the CT, including because of difficulties obtaining anaesthetic support.

#### Interventional Radiology

7.8% (36/459) underwent an IR procedure, the reasons for which are shown in Table 7.

**Table 7:** Reasons for interventional radiology procedures (answers may be multiple)

Reason for interventional radiology	Number of patients
Haemostasis not achieved endoscopically	16
Diagnosis on CTA	18
Haemodynamically unstable, no bleeding on CTA	7
Haemodynamically unstable, CTA not performed	4
TIPSS	2

11 were haemodynamically stable at the time of the IR procedure. In 89% (32/36), the IR procedure was within an appropriate time for the patient’s condition. There were six re-bleeds post IR and two complications (one intestinal necrosis and one coil misplacement).

18 patients had intervention performed with 16 embolisations. Whether treatment is performed or not, a re-bleed plan should be documented. This occurred in 65% (21/32).

TIPSS only accounted for two IR interventions. This is explained by its availability in very few centres, combined with the equal sampling across all hospitals along with difficulties in obtaining complete case notes from two separate institutions for patients who had an inter-hospital transfer for TIPSS resulting in the exclusion of a number of TIPSS cases.

A further 21 patients (6.3%) did not undergo an IR procedure but should have done in the opinion of the reviewing panel. In total, 14.1% did or should have undergone an IR procedure.

#### Equipment Replacement Programme

42% (78/186) of hospitals had a formal high-cost equipment programme for imaging equipment including CT scanners and angiography. It has been recommended by The European Society of Radiology that all hospitals should have an equipment replacement programme which looks forward a minimum of 5 years and is reviewed annually [11]. Equipment older than 10 years of age is no longer state-of-the-art and replacement is recommended.

#### Surgery

There has been a 50% reduction in emergency laparotomies over the past 10 years in England and Wales according to the National Emergency Laparotomy Audit (NELA) 2015 with approximately 600 annually laparotomies for GI bleeding across 205 hospitals [12]. There are understandable concerns that surgical trainees will not get sufficient exposure to attain emergency competency and that established consultants will not be able to maintain that competency.

6% (36/586) underwent surgery with 21/36 for uncontrolled bleeding. Suture controls of a peptic ulcer (14), large (8) or small bowel (7) resection were the commonest. Six were for bleeding following the IR therapy and three had evidence of peritonitis. The reviewers found that in 20/35, there was no discussion with an Interventional Radiologist despite the majority being considered suitable for IR by the reviewing surgeons. In nine, this was because no IR was available on-site or was not available out of hours.

#### Re-bleeding

23.2% (138/595) in this cohort receiving 4 or more units of blood had one or more episode of re-bleeding. Re-bleeding occurred in a similar proportion of upper and lower GI bleeds (22.5 vs. 25.4% respectively). In-patients with a GI bleed were more likely to re-bleed than new admissions (27.3 vs. 19.2%, respectively). Despite the high rate of re-bleeding, a documented plan in the event of a re-bleed was commonly not considered with re-bleed plans at OGD, IR and Surgery of 58.4, 65 and 38%, respectively.

#### Outcome and Quality of Care

The significant physiological insult of severe GI bleeding along with uncertainty as to when it is safe to discharge patients is reflected in the length of hospital stay for new admissions with a GI bleed. Over half of the patients stayed in for 8 days or more, 20% remained in hospital for more than 18 days and 10% were still in hospital a month after their admission.

The methodology used which oversamples smaller volume units relative to their exposure to the overall prevalence of GI bleeding may exaggerate numerical outcome data which should be considered with some caution. This probably accounts for the slightly higher mortality rate in this cohort of 23.7% (142/618) compared to the unskewed mortality rate of 20.2% for those receiving 4 or more units of blood in the total ICD10 coded population. Mortality was similar for non-variceal upper GI bleeds (21.5% 77/358) and lower GI bleeds 20% (28/138). Mortality for variceal (32% 16/50) and those without a diagnosis (29% 21/72) was also similar. 37.7% (89/245) of patients who developed a GI bleed as a complication of an admission for another condition died compared to 14.4% (52/370), where GI bleeding was the cause of the admission.

Overall, the case reviewers considered that 44.1% (210/476) had care that could be categorised as Good Practice (Appendix). In 45% (214/476), there was room for improvement in clinical care and in 18.5% (88/476) organisational factors required improvement. In 4.4% (21/479) care was less than satisfactory.

## Conclusions and Recommendations to Improve Care

The report makes 26 recommendations to improve the quality care of patients with GI bleeding, including six principle recommendations. Many of the issues identified in the report will be familiar to all those who regularly manage GI bleeds, but providing the evidence to support change can be difficult. Quality of care studies can provide that evidence and drive change.

The six principle recommendations were as follows:

1. Patients with acute GI bleeds should only be admitted to hospitals with 24/7 on-site endoscopy and surgery and on-site or formally networked IR services.

A 24/7 on-site recommendation for IR services could not be justified as the current level of provision was so low that such a recommendation would have meant restricting acute GI bleed admissions to less than a quarter of hospitals, which would likely have a negative effect on overall care.

2. Hospitals that do not admit patients with GI bleeds must have 24/7 access to endoscopy, interventional radiology and GI bleed surgery for patients who develop a GI bleed whilst as an in-patient for another condition by either an on-site service or a formal network.
3. The traditional separation of care for upper and lower GI bleeding in hospitals should stop. All acute hospitals should have a single integrated service for all GI bleeds.

The lack of specificity of presenting features documented in the report means that the commonest current arrangement in the UK results in two separate teams is managing a mixture of upper and lower GI bleeds. There are opportunities to improve care by sharing nursing and medical expertise in a single service.

4. All patients who present with a major^2^ upper or lower GI bleed, either on admission or as an in-patient, should be discussed the consultant on-call for the GI bleed service within 1 hour of the diagnosis of a major bleed.
5. The ongoing management for patients with a major bleed is the responsibility of the duty consultant for the GI bleed service; to ensure timely investigation and treatment to stop bleeding and reduce unnecessary blood transfusion.
6. All patients with a GI bleed must have a clearly documented re-bleed plan agreed at the time of each diagnostic or therapeutic intervention

In England, Wales and Northern Ireland, Interventional Radiology Services for GI bleeding are limited in their on-site availability with only 26% of hospitals who admit GI bleeds having a 24/7 on-site service. Approximately, a half of hospitals have not made arrangements, in the form of formal networks, to compensate for this deficiency despite established national guidelines identifying the importance of IR in management algorithms. Peer review of case notes demonstrates IR is underused and often not considered, and CT has a much lower diagnostic rate that suggested by well-controlled clinical studies. Greater efforts are required to integrate IR into locally agreed management plans for upper and lower GI bleeding, preferably as part of a single integrated GI bleed unit.

## Appendix

NCEPOD quality of care grading system

*Good practice* A standard that you would accept from yourself, your trainees and your institution.

*Room for improvement* Aspects of *clinical care* that could have been better.

*Room for improvement* Aspects of *organisational care* that could have been better.

*Room for improvement* Aspects of both *clinical and organisational* care that could have been better.

*Less than satisfactory* Several aspects of *clinical and/or organisational* care that were well below that you would accept from yourself, your trainees and your institution.

*Insufficient data* Insufficient information submitted to NCEPOD to assess the quality of care.

## Footnotes

1 NCEPOD is an independent body. This project was undertaken as part of the Clinical Outcome Review Programme into Medical and Surgical Care which is funded by the Healthcare Quality Improvement Partnership on behalf of NHS England, NHS Wales, the Northern Ireland Department of Health, Social Services and Public Safety (DHSSPS), the States of Jersey, Guernsey, and the Isle of Man. Co-operation with national confidential enquiries is a statutory requirement for UK doctors and hospitals. Under NHS Act 2006 NCEPOD has been granted Section 251 approval by the Secretary of State for Health which permits the use of patient identifiable data without patient consent. All data are anonymised by NCEPOD non-clinical staff prior to review.

2 Local guidelines will need to define a major bleed pending National or International Guidelines/consensus.
